# IL-6 Negatively Regulates IL-22R*α* Expression on Epidermal Keratinocytes: Implications for Irritant Contact Dermatitis

**DOI:** 10.1155/2019/6276254

**Published:** 2019-10-29

**Authors:** Benjamin Frempah, Lerin R. Luckett-Chastain, Randle M. Gallucci

**Affiliations:** Department of Pharmaceutical Sciences, University of Oklahoma Health Sciences Center, 1110 N. Stonewall Avenue, Oklahoma City, OK 73117, USA

## Abstract

Irritant Contact Dermatitis (ICD) is characterized by epidermal hyperplasia and inflammatory cytokine release. IL-6 has been shown to be involved in the pathogenesis of ICD; however, the involvement of the IL-22/IL-22R*α* axis and its relation to IL-6 in the inflammatory response following irritant exposure are unknown. Using a chemical model of ICD, it was observed that mice with a keratinocyte-specific knockout of IL-6R*α* (IL-6R*α*^Δker^) presented with increased inflammation and IL-22R*α* and IL-22 protein expression relative to WT following irritant exposure, indicating that IL-6R*α* deficiency in epidermal keratinocytes leads to the upregulation of IL-22R*α* and its ligand during ICD. Furthermore, it was shown that IL-6 negatively regulates the expression of IL-22R*α* on epidermal keratinocytes. This effect is functional as the effects of IL-22 on keratinocyte proliferation and differentiation were markedly reduced when keratinocytes were pretreated with IL-6 prior to IL-22 treatment. These results show that IL-6 modulates the IL-22/IL-22R*α* axis in the skin and suggest that this occurrence may be associated with the increased epidermal hyperplasia and exacerbated inflammatory response observed in IL-6R*α*^Δker^ mice during ICD.

## 1. Introduction

IL-22 is a proinflammatory cytokine that belongs to the IL-10 family of cytokines [1]. IL-22 directly acts on epithelial cells by binding to a heterodimer receptor complex made up of the IL-22 receptor alpha (IL-22R*α*) and the IL-10 receptor beta (IL-10R*β*) [2, 3]. IL-22R*α* is expressed primarily by epithelial cells of the skin, kidneys, and lungs, while IL-10R*β* is relatively ubiquitous [3]. Thus, only cells that bear IL-22R*α* can respond to the effects of IL-22 [4, 5]. IL-22 is produced by several immune cell types including Th1 [6], Th17 [7, 8], Th22 [9, 10], *γδ* T, NKT, and innate lymphoid cells [11]. Since epidermal keratinocytes bear IL-22R*α* [4], IL-22 has been shown to promote keratinocyte proliferation [12] while inhibiting its differentiation [4, 13]. The role of IL-22 has also been widely reported in several inflammatory skin diseases. For instance, high serum levels of this cytokine have been shown to correlate with poor disease prognosis in psoriasis [13], and psoriatic skin has also been shown to express higher levels of IL-22 mRNA relative to normal skin from controls [14, 15]. IL-22 also promotes epidermal barrier disruption and pruritus and has been reported to play a role in the pathogenesis of atopic dermatitis [5, 16].

IL-6 is a pleiotropic cytokine with proinflammatory, anti-inflammatory, and immune modulating functions on numerous cell and tissue types [17, 18]. The IL-6 signaling complex comprises IL-6, IL-6 receptor alpha (IL-6R*α*), and the ubiquitous signal transduction unit, gp130 [18, 19]. Homodimerization of the receptor complex can lead to the activation of STAT3 and STAT1, as well as the ERK and PI3K pathways [19]. IL-6 is produced by numerous immune cells including T cells, macrophages, neutrophils, and nonimmune cells like keratinocytes and fibroblast in the skin [20, 21]. IL-6 has also been shown to affect the function of cells in the skin [21]. For example, IL-6 promotes keratinocyte proliferation and migration [22, 23] and increases fibroblast proliferation [24].

Irritant Contact Dermatitis (ICD) is an inflammatory response of the skin to chemical or physical irritants and is characterized by epidermal hyperplasia, inflammatory cell influx into lesional skin, and inflammatory cytokine release including IL-6 [25, 26]. While it is known that IL-6 confers a protective effect to the skin during ICD [20], the role of IL-22 is unknown. It has been reported that IL-22 promotes epidermal barrier disruption and also promotes epidermal hyperplasia [11] which are characteristics of ICD; however, the role of this cytokine in this phenomenon is unknown.

The purpose of this study was to determine the relationship between IL-6 and the IL-22/IL-22R*α* system in epidermal keratinocytes. The relationship between the IL-22/IL-22R*α* axis and epidermal hyperplasia during ICD was also explored. Results presented herein show that IL-6 treatment decreases IL-22R*α* expression on epidermal keratinocytes. Furthermore, the effects of IL-22 on keratinocyte differentiation and proliferation were diminished in the presence of IL-6. These results provide useful insight on the role of IL-6 and IL-22 during ICD and also begin to shed light on how IL-6 influences the expression and function of other cytokines during skin inflammation.

## 2. Results

ICD is characterized by epidermal hyperplasia and increased inflammatory cytokine release [27]. To determine how IL-6R*α* function in epidermal keratinocytes influences epidermal thickening during ICD, mice with a keratinocyte knockout of IL-6R*α* (IL-6R*α*^Δker^) and littermate controls (WT) were exposed to benzalkonium chloride (BKC), a well-characterized irritant of human and murine skin, and acetone control for seven (7) consecutive days.

**Figure 1:**
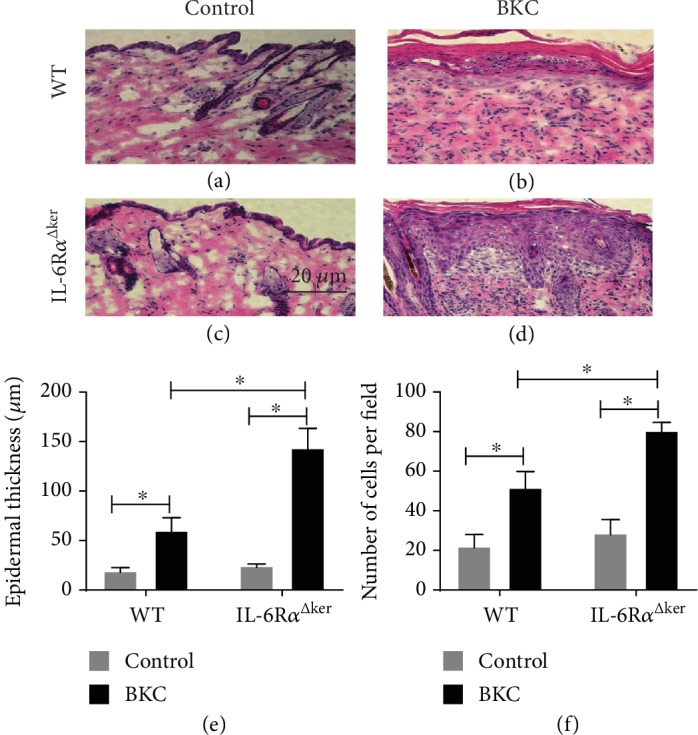
IL-6R*α*^Δker^ mice present with increased epidermal hyperplasia following irritant exposure. Loss of the IL-6R*α* in keratinocytes promotes epidermal hyperplasia during irritant contact dermatitis. WT and IL-6R*α*^Δker^ mice were exposed to BKC or control for seven (7) consecutive days to induce ICD. 24 hours after irritant exposure, 8 mm biopsies of lesional skin were collected and embedded in O.C.T. compound for histological analysis. Skin samples were cross-sectioned and then hematoxylin and eosin (H&E) stained. Representative H&E stains from WT (a, b) and IL-6R*α*^Δker^ (c, d) are shown. Quantification of epidermal thickness (e) and cells per field (f) as determined by ImageJ (NIH) is presented. Data are mean ± SD. ^∗^Significantly different from WT (*p* ≤ 0.05, *n* = 15 mice/treatment/genotype).

Quantitative analysis of hematoxylin and eosin (H&E) staining from lesional skin revealed that IL-6R*α*^Δker^ mice presented with significantly increased epidermal hyperplasia relative to WT after seven (7) days of BKC exposure (Figures 1(a)–1(e)).

**Figure 2:**
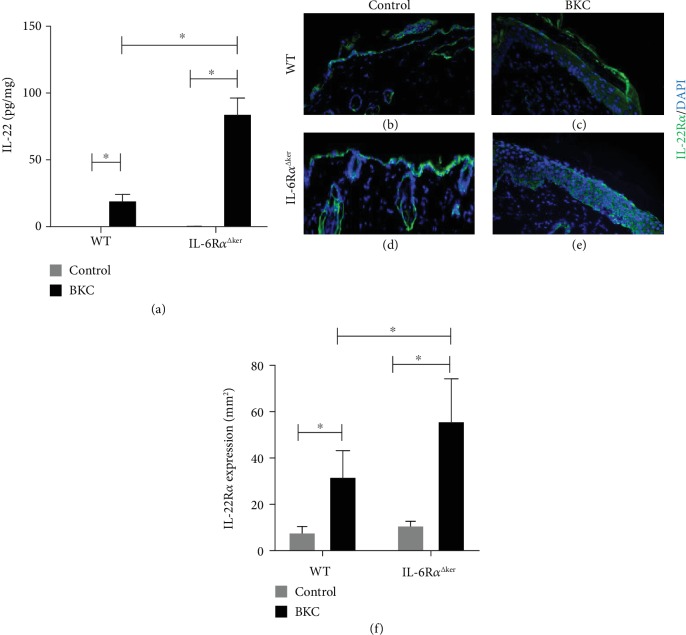
IL-6R*α*^Δker^ mice express higher levels of IL-22R*α* and IL-22 in lesional skin. Irritants induce higher expression of IL-22 and IL-22R*α* in mice with a keratinocyte-specific knockout of IL-6R*α*, IL-6R*α*^Δker^, and WT mice were exposed to BKC or control for 7 consecutive days. Lesional skin was harvested from each genotype and IL-22 protein expression was determined by Luminex immunoassay (a). Immunohistochemical analysis of lesional skin from irritant-exposed mice, stained for the expression of IL-22R*α* (green), and nuclear staining DAPI (blue). Representative images from WT (b, c) and IL-6R*α*^Δker^ (d, e). Quantification of IL-22R*α* expression as determined by ImageJ (NIH) is presented (f). Data are mean ± SD. ^∗^Significantly different from WT (*p* ≤ 0.05, *n* = 15 mice/treatment/genotype).

Overexpression of IL-22 in the skin has been shown to promote epidermal hyperplasia [28], and IL-6 is well known to affect skin function and inflammation. However, it is still unclear if there is a link between IL-6 function and the expression of IL-22 and its receptor. To evaluate this, IL-22 protein in lesional skin from IL-6R*α*^Δker^ relative to WT mice was assessed via Luminex assay following exposure to the irritant BKC as described [29]. Irritant exposure itself increased IL-22 expression, and keratinocyte-specific IL-6R*α* deficiency increased its expression nearly fourfold (Figure 2(a)). Additionally, immunohistochemistry revealed higher levels of IL-22R*α* protein in lesional skin from IL-6R*α*^Δker^ mice relative to WT (Figures 2(b)–2(f)).

**Figure 3:**
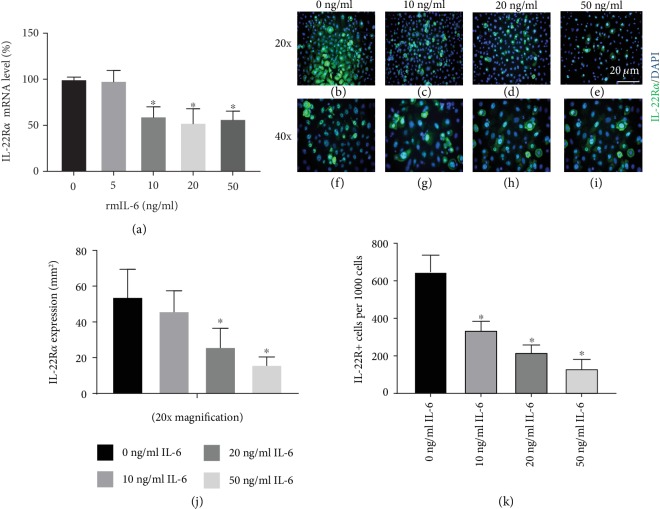
IL-6 negatively regulates IL-22R*α* expression on epidermal keratinocytes. Primary keratinocytes from IL-6KO mice were treated with rmIL-6 for 4/24 hours (mRNA/protein expression) at the indicated concentrations. The expression of IL-22R*α* mRNA was analyzed and normalized to 28S ribosomal RNA as control (a). IL-6KO keratinocytes were grown to confluency on multichamber slides. Immunohistochemical analysis of keratinocyte culture stained for the expression of IL-22R*α* (green), and nuclear staining DAPI (blue). Representative fluorescent images are shown at 20x (b–e) and 40x (f–i). Quantification of IL-22R*α* expression as determined by ImageJ (NIH) is presented (j and k). Data are mean ± SD. ^∗^Significantly different from 0 ng/ml rmIL-6 (*p* ≤ 0.05, *n* = 3 separate experiments).

To investigate further the modulation of IL-22 function by IL-6R*α*, cultured epidermal keratinocytes from IL-6-deficient (IL-6KO) mice were incubated with recombinant mouse IL-6 (rmIL-6) in a dose-dependent (0-50 ng/ml) manner and total mRNA was isolated. We have found through our multiple publications that the biologically active concentration of IL-6 is above 5 ng/ml and less than 50 ng/ml in culture by conducting dose-response experiments [22]; thus, we employed IL-6 concentrations of 0-50 ng/ml. Treatment with higher doses of rmIL-6 (10-50 ng/ml) led to a significant reduction in the expression of IL-22R*α* (Figure 3(a)).

Immunohistochemical analysis also revealed that treating epidermal keratinocytes with rmIL-6 led to a reduction in the expression of IL-22R*α* protein (Figures 3(b)–3(k)).

**Figure 4:**
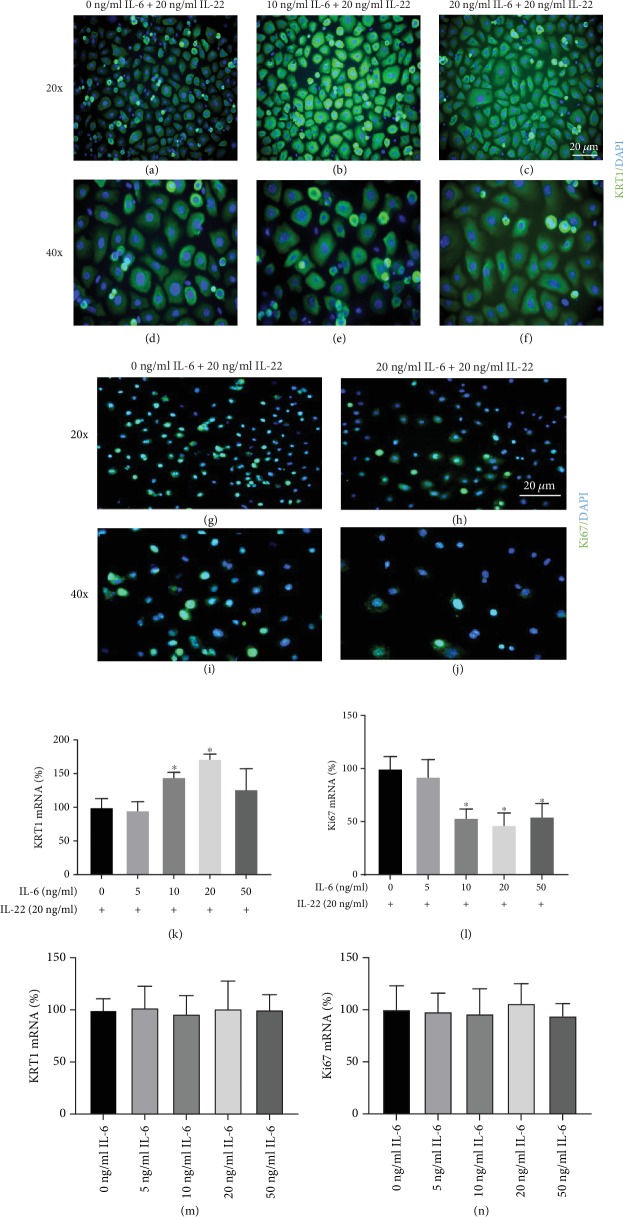
IL-6 reduces the effect of IL-22 on keratinocyte differentiation and proliferation. Primary keratinocytes were pretreated with rmIL-6 for 24 hours followed by exposure to 20 ng/ml rmIL-22 for 24 hours (a–f and g–j) or 4 hours (k and l). Immunohistochemical analysis of IL-6KO keratinocyte expression of KRT1 (a–f) or Ki67 (g–j) (green) protein and nuclear staining with DAPI (blue). Representative images are shown. KRT1 expression (k) and Ki67 (l) mRNA expression were analyzed by RT-PCR. Keratinocytes were exposed to rmIL-6 alone in a dose-dependent manner for 4 hours, and KRT1 (m) and Ki67 (n) mRNA were analyzed by RT-PCR. Data are mean ± SD. ^∗^Significantly different from 0 ng/ml rmIL-6 (*p* ≤ 0.05, *n* = 4 separate experiments).

IL-22 has multiple effects on keratinocyte functions including promoting proliferation and inhibiting differentiation [4]. To determine whether IL-6 will have an influence on the functional effect of IL-22 on epidermal keratinocytes, primary IL-6KO keratinocytes were treated with rmIL-6 in a dose-dependent manner for 24 hours. This was followed by treatment with 20 ng/ml of rmIL-22 for 24 hours. Higher expression of keratin 1 (KRT1), a marker of keratinocyte differentiation [30], was observed when keratinocytes were treated with rmIL-6 prior to rmIL-22 treatment (Figures 4(a)–4(f)) indicating increased differentiation.

As well, RT-PCR revealed that pretreatment with rmIL-6 increased the expression of KRT1 mRNA in keratinocytes in response to IL-22 (Figure 4(k)). Interestingly, no effects of IL-6 on filaggrin expression was observed in the present study (results not shown).

Further, the effect of IL-6 on IL-22-induced keratinocyte proliferation was analyzed via the expression of the Ki67 proliferation marker. IL-22-induced keratinocyte Ki67 protein (Figures 4(g)–4(j)) and mRNA (Figure 4(l)) were decreased following rmIL-6 pretreatment. No effects on KRT1 and Ki67 mRNA were observed when keratinocytes were exposed to IL-6 alone (Figures 4(m) and 4(n)).

## 3. Discussion

The protective role of IL-6 in the context of the skin has been reported. In fact, during skin inflammation and wound healing, IL-6 has been shown to promote skin repair and regeneration [31, 32]. The effect of IL-6 on keratinocyte function has also been well reported where IL-6 has been shown to promote migration and proliferation [22, 23, 33]. The effect of IL-6 on the IL-22-IL-22R*α* system in the context of the skin inflammation is, however, not well documented. Herein, we show that lesional skin from mice with a keratinocyte-specific knockout of the IL-6R*α*, which are known to display increased skin irritation [29], presented with increased expression of IL-22R*α* following irritant exposure. Consistent with these results, primary keratinocytes from IL-6KO mice pretreated with IL-6 displayed the downregulation of the expression of IL-22R*α*.

IL-22R*α* expression on epithelial cells plays an essential role in several inflammatory diseases. IL-22 is a proinflammatory cytokine that promotes skin inflammation and epidermal hyperplasia [34, 35]. IL-6R*α*^Δker^ mouse skin displayed significantly higher epidermal hyperplasia and immune cell infiltration relative to WT following seven-day exposure to the irritant BKC (Figure 1). These results were consistent with an earlier study where IL-6R*α*^Δker^ mice exposed to jet propellant 8 fuel and BKC for a three-day period presented with an exaggerated inflammatory response [29]. Also, higher expression of IL-22R*α* and IL-22 protein expression were observed in lesional skin from IL-6R*α*^Δker^ relative to WT mice (Figure 2). Increased expression of IL-22R*α* on keratinocytes promotes the responsiveness of keratinocytes to the effect of IL-22 [36]. Thus, increased expression of both IL-22 and IL-22R*α* on lesional skin from IL-6R*α*^Δker^ mice during ICD might be associated with the increased inflammation and epidermal hyperplasia observed in these mice. In contrast to IL-22, IL-6 has been reported to confer a protective effect to the skin during inflammation [20]. The finding that the loss of IL-6R*α* in epidermal keratinocytes led to enhanced inflammation and epidermal thickening during ICD appears to suggest that IL-6 may be associated with modulating the IL-22-IL-22R*α* system in keratinocytes. Indeed, results from *in vitro* experiments revealed that IL-6 negatively regulates the expression of IL-22R*α* on epidermal keratinocytes (Figure 3). Evidence for the dynamic regulation of the expression of IL-22R*α* on epidermal keratinocytes exists where for instance IFN-*α* enhances IL-22R*α* expression on keratinocytes [37]. However, the negative regulation of the expression of IL-22R*α* on keratinocytes has not been previously demonstrated. IL-6-mediated downregulation of the expression of IL-22R*α* on keratinocytes indicates that one way IL-6 might act in a protective manner in skin is by modulating the inflammatory role of the IL-22-IL-22R*α* system.

Normal keratinocyte differentiation is necessary for epidermal barrier integrity [38]. IL-22 inhibits keratinocyte differentiation and therefore impairs epidermal barrier integrity [28, 39]. In the presence of rmIL-6, however, the effects of IL-22 on keratinocyte function are diminished. Specifically, the effect of IL-22 on keratinocyte differentiation was diminished when keratinocytes were treated with rmIL-6 prior to IL-22 exposure (Figure 4). Thus, IL-6 might play a role in promoting the integrity of the epidermal barrier. Indeed, IL-6 is known to be involved in promoting epidermal barrier integrity [40] and given the findings presented herein, IL-6 possibly promotes epidermal barrier integrity via reducing the function of IL-22 on keratinocytes. IL-6 alone did not have any effects on KRT1 levels in the present study (Figure 4). The results presented in this work suggest that the effect of IL-6 on IL-22R*α* during ICD is an active mechanism of the immune system to control skin barrier disruption. In WT skin, increased IL-6 expression reduces IL-22R*α* expression which in turn helps to reduce epidermal hyperplasia.

Despite the well-characterized effects of IL-22 on epidermal keratinocytes, very little is known about the regulation of IL-22R*α* expression on these cells. It is known that IL-22R*α* expression is upregulated following stimulation of human skin cell lines with rIFN-*γ* [15]. Thus, decreased expression of IFN-*γ* in the skin could lead to a downregulation of IL-22R*α* on epidermal keratinocytes. Also, it has been reported that the concentration of IFN-*γ* is inversely correlated with increased IL-6 signaling [41]. Therefore, the observed negative regulation of IL-22R*α* by IL-6 might be associated with the effects of IL-6 on IFN-*γ*. Further studies are required to better characterize the intracellular mechanisms involved in the negative regulation of IL-22R*α* expression by IL-6.

Increased proliferation of keratinocytes has been shown to promote epidermal hyperplasia and is referred to as acanthosis [42]. In the context of skin inflammatory diseases like psoriasis and allergic contact dermatitis, increased proliferation of keratinocytes has been linked to increased acanthosis, a prominent debilitating result of these diseases [43]. The present data that pretreatment with rmIL-6 reduces the effect of IL-22 on keratinocyte proliferation suggest that IL-6 might play a role in modulating epidermal hyperplasia during ICD by reducing the increased proliferation induced by IL-22. Indeed, IL-6R*α*^Δker^ mice presented with higher epidermal hyperplasia relative to WT after ICD (Figure 1 and [29]). Further, IL-22 promotes the expression of proinflammatory cytokines including IL-1, TNF-*α*, and GM-CSF, all of which have been shown to be highly expressed in lesional skin following irritant exposure [25] and are well known to be involved in the pathology of psoriasis. Previous work from this group has revealed that IL-6 alone does not affect keratinocyte proliferation/viability [22]. Indeed, using both BrdU and WST, it was observed that IL-6 alone did not affect either way at various doses. It is also interesting to state that in this same study, this group did not observe any mitogenic property for IL-6. Thus, the observed effects cannot be attributed to the hyperproliferation of keratinocytes following exposure to IL-6. The effects on increased keratinocyte proliferation cannot be ruled out in the in vivo setting. Indeed, the observed increased IL-22R*α* staining in lesional skin from IL-6R*α*^Δker^ mice could also be a function of the increased proliferation of keratinocytes as seen in Figures 1 and 2. To rule this out, it was confirmed at the cellular level using in vitro experiments that IL-6 negatively regulates the expression of IL-22R*α* (Figure 3) and thus confirming the results observed in IL-6R*α*^Δker^ mice. Taken together, these results suggest that IL-6 acts to ameliorate epidermal hyperplasia during skin inflammation by reducing IL-22-induced keratinocyte proliferation.

The role of IL-6 in the pathogenesis of a host of inflammatory skin diseases has been well documented. Indeed, high levels of IL-6 protein have been observed in psoriatic skin with increased levels of this cytokine leading to poor prognosis [44, 45]. Also, interrupting IL-6 signaling has been shown to improve atopic dermatitis suggesting that IL-6 contributes to its pathogenesis [46]. Several reports have also implicated the IL-22/IL-22R*α* system in the pathogenesis of psoriasis and atopic dermatitis. As mentioned previously, these conditions are associated with epidermal hyperplasia. It is interesting to note that the relationship between IL-6 and the IL-22/IL-22R*α* system in the context of skin inflammation is unknown. Results from this work suggests that IL-6 might play a role in regulating the IL-22/IL-22R*α* system during skin inflammation.

## 4. Summary and Conclusions

Based on the evidence presented herein, we propose that during ICD, IL-6 limits epidermal hyperplasia *via* reducing the responsiveness of epidermal keratinocytes to IL-22. Specifically, IL-6 acts to limit the functional role of IL-22 on epidermal keratinocytes through the reduced expression of IL-22R*α*. Taken together, the results of this study highlight a potential role of the IL-22-IL-22R*α* system in the pathogenesis of ICD and reveal a previously unknown role of IL-6 in regulating the effects of the IL-22-IL-22R*α* system. These findings also provide useful insight into the exacerbated response of murine skin to irritants in the absence of IL-6/IL-6R*α* in epidermal keratinocytes. While the current studies demonstrate that IL-6 negatively regulates the expression of IL-22R*α* on epidermal keratinocytes, there are limitations with the method used. Indeed, quantification of the expression of IL-22R*α* and KRT1 expression with flow cytometry and/or ELISA will help better characterize the effects of IL-6 on the IL-22/IL-22R*α* system. Future studies using such methods are therefore warranted.

Even though the results presented in this study are specific to irritant contact dermatitis, they may lend insight into pathologies involving inflammation and keratinocyte dysregulation such as psoriasis.

## 5. Methods

### 5.1. Mice

Mice with a keratinocyte-specific knockout of IL-6R*α* (IL-6R*α*^Δker^) were generated by crossing Il6ra^fl/fl^ to K14CreERT mice, and a breeding colony was maintained by mating Il6ra^fl/fl^ mice with Il6ra^fl/fl^ K14Cre^Tg/wt^ mice. All mice were bred and maintained at the rodent barrier facility of the University of Oklahoma Health Sciences Center (OUHSC). All animal experiments were conducted in accordance to protocols approved by the Institutional Animal Care and Use Committee (IACUC) of the OUHSC (protocol ID: OUHSC IACUC 16-083).

### 5.2. Dermal Irritant Exposure

Mice at the age of 6-8 weeks were used for ICD experiments. To induce dermatitis, we utilized a murine model of ICD as previously described [20, 47]. Mice were sedated with isoflurane, and ~5 cm of hair was removed via shaving on the dorsal side of the animals 24 hours prior to initial irritant exposure. 50 *μ*l of 2% benzalkonium chloride (BKC) or acetone control was applied to the shaved back skin of mice daily for seven (7) consecutive days. Mice were euthanized 24 hours after the last day of irritant exposure, and lesional skin was harvested and used for histological and Luminex immunoassays.

### 5.3. Primary Keratinocyte Culture

Primary keratinocytes were isolated from 2- to 3-day-old IL-6-deficient mice according to the method described by Lichti et al. [48]. Briefly the skin was isolated and floated overnight in dispase at 4°C. The epidermis was separated from the dermis and plated on collagen-coated 60 mm plates/8-well chamber slides and cultured in a humidified incubator with 5% CO_2_ at 37°C; fresh media were replenished every 48 hours. Recombinant mouse IL-22 protein (BioLegend, #576204) was used at a concentration of 20 ng/ml.

### 5.4. Skin Histology and Immunohistochemistry

Cryosections were prepared as 8 mm skin cross-sections and hematoxylin and eosin (H&E) stained. Digital images of the skin histopathology were acquired with a Leica DM4000B microscope (Leica Microsystems, Buffalo Grove, IL). Analysis of epidermal thickness was conducted with ImageJ (NIH). Immunohistochemistry sections were fixed with 4% paraformaldehyde and stained essentially as previously described [49]. The following bound primary antibodies—IL-22R*α* (clone 305405, concentration used: 1 in 400), Invitrogen; KRT1 (ab93652, concentration used: 1 in 250), Abcam; and Ki67 (ab15580, concentration used: 1 in 250), Abcam—were detected by incubation with Alexa Fluo 488 conjugated secondary antibody (Invitrogen, concentration used: 1 in 500), followed by counterstaining with DAPI (Vector Labs, Burlingame, CA), and visualized under fluorescence using a Leica DM4000B microscope.

### 5.5. RT-PCR

Total RNA was isolated from cultured keratinocytes using the TRI reagent according to the manufacturer's instructions. RNA was used to synthesize cDNA as previously described [22]. Quantitative real-time RT-PCR was performed on Applied Biosystems StepOnePlus (Thermo Fisher Scientific, Waltham, MA). Gene expression was normalized to 28S using the ΔΔCT method [50]. Murine primers were designed according to published sequences, and synthesized by Invitrogen (Waltham, MA). The primer sequences for the specific genes are listed below:

- 28S
  - Forward: 5′-GGCAACAACACATCATCAG-3′
  - Reverse: 5′-CAGTACGAATACAGACCG-3′
- IL-22R*α*
  - Forward: 5′-CTACGTGTGCCGAGTGAAGA-3′
  - Reverse: 5′-AAGCGTAGGGGTTGAAAGGT-3′
- Keratin 1
  - Forward: 5′-CCAGTTCTCCTCTGGATCGCAG-3′
  - Reverse: 5′-GATCTTCCAGTGGGATCTGTGTCCA-3′
- Ki67
  - Forward: 5′-CTGCCTGCGAAGAGAGCATC-3′
  - Reverse: 5′-AGCTCCACTTCGCCTTTTGG-3′

### 5.6. Statistical Analysis

All experiments were replicated, and representative findings are presented. Statistical significance between groups were determined by two-way ANOVA followed by Sidak's multiple comparison test. *p* values of 0.05 or less were considered statistically significant.

## Data Availability

No data were used to support this study.

## Abbreviations

IL-6R*α*^Δker^: Keratinocyte-specific IL-6R*α* knockout
ICD: Irritant contact dermatitis
BKC: Benzalkonium chloride
NKT: Natural killer T cells
*γδ* T: Gamma delta T cells.
